# Use of Termites by Farmers as Poultry Feed in Ghana

**DOI:** 10.3390/insects10030069

**Published:** 2019-03-13

**Authors:** Hettie Arwoh Boafo, Siegfried Affedzie-Obresi, Dossou Séblodo Judes Charlemagne Gbemavo, Victor Attuquaye Clottey, Emmanuel Nkegbe, Gabriel Adu-Aboagye, Marc Kenis

**Affiliations:** 1 CABI, CSIR Campus, P.O. Box CT 8630, Cantonments, Accra, Ghana; h.boafo@cabi.org (H.A.B.); v.clottey@cabi.org (V.A.C.); 2 Animal Research Institute of the Council for Scientific and Industrial Research, P.O. Box AH20, Achimota, Ghana; obresis@yahoo.com (S.A.-O.); kwakunkegbe@yahoo.com (E.N.); gaduaboagye@gmail.com (G.A.-A.); 3 Laboratoire de Biomathématiques et d’Estimations Forestiéres (LABEF), Faculté des Sciences Agronomiques (FSA), Université d’Abomey Calavi, Cotonou 04 BP 1525, Benin; cgbemavo@yahoo.fr; 4 Unité de Biostatistique et de Modélisation (UBM), Faculté de Sciences et Techniques (FAST) Université Nationale des Sciences, Technologies, Ingénierie et Mathématiques (UNSTIM), Dassa-Zounm BP 14, Benin; 5 CABI. Rue des Grillons 1, CH-2800 Delémont, Switzerland

**Keywords:** termites, *Macrotermes*, *Trinervitermes*, *Odontotermes*, Ghana, poultry feed

## Abstract

The aim of the study was to gather information on the use of termites by farmers as feed for indigenous poultry in Ghana and factors affecting its use. We conducted surveys in four regions in Ghana to collect information, by the administration of questionnaires, on the use of termites as poultry feed, termite species collected, species not used and collection methods. Samples of termite species mentioned were collected and identified to the genus level. Twenty-three percent and 19% of farmers mentioned that termites are always or often used to feed poultry whereas 11% never use termites. A binomial regression analysis showed that their utilization was affected by region, sex, education, farm size and income. Termites collected belonged to eight genera, the main ones being *Macrotermes*, *Trinervitermes* and *Odontotermes*. Five collection methods are used to obtain termites and involve either breaking mounds or using containers as traps. Collection methods vary with species and region and the abundance of termite genera varies with season. Farmers identified some species as poisonous to poultry. Termites are important in indigenous poultry production because they are a readily available protein source for local farmers. However, better collection methods need to be developed to aid their optimal use.

## 1. Introduction

The use of termites by man is a common practice in Africa, Asia, Latin America and Australia. Figueirêdo et al. [1] recorded the use of 43 species of termites belonging to four families being used by humans as food or feed for livestock. Termites are known to be nutritious for both humans and animals [2,3,4,5]. The use of termites as human food is well documented [1,5,6,7,8,9,10,11]. In contrast, data on termites as animal feed are scarce. In West Africa, information is mostly restricted to anecdotal reports in general articles and reviews [12,13,14] or to technical notes and unpublished theses [15,16,17,18,19]. Nevertheless, termites are commonly used to feed poultry. A recent publication highlighted that, in Burkina Faso, about 78% of the farmers feed termites to their poultry at least occasionally [20]. Termites are an essential feed for small African poultry farmers that have no other affordable source of protein at their disposal and, therefore, methods to enhance their use are most desirable.

In West Africa, termites are collected from chippings of termite mounds or by trapping, using inverted pots or baskets filled with organic matters and placed in the vicinity of termite nests [16,17,18]. In general, these systems do not allow collecting a large number of termites and, thus, these are often fed to chicks and guinea fowl keets only, for which protein intake is critical for their survival. Availability and accessibility of termites to farmers in different regions and different seasons vary [20]. At certain seasons, farmers travel long distances to harvest termites [14]. In a survey in Benin [15], farmers reported that some species of termites are toxic to birds and therefore unsuitable as feed. *Noditermes* species caused mortalities in two poultry species while *Trinervitermes* species were safe. In Burkina Faso, it was reported that some species of *Cubitermes* are toxic to chicks of domestic fowls but not to guinea fowl and ducks [19]. 

In Ghana, to the best of our knowledge, no such documentation exists and yet verbal communication with farmers practicing indigenous poultry rearing shows the extensive use of termites in their feeding activities. It has, therefore, become imperative to gather knowledge on the practice to allow the assessment of the different techniques in order to disseminate the most efficient ones and improve the availability of protein feed among smallholder farmers. The study was conducted through two large-scale surveys in four regions in Ghana, to access the utilization of termites as poultry feed in the country, determine factors that influence the use of termites, identify the termite species used as feed and the toxic species, assess the availability at different seasons and describe the methods used in obtaining termites. 

## 2. Materials and Methods 

### 2.1. Region of Investigation

The investigation on the use of termites as feed in traditional poultry production was conducted in four regions of Ghana, i.e., Volta, Northern, Upper East and Upper West. These regions were purposefully selected due to their dominance in indigenous poultry production. 

The Volta region is in the southern half of the country fringing the eastern border. The vegetation varies from coastal grassland, mangrove swamps, guinea savannah, to mountainous wooded savannah, marked by two annual rainfall seasons. The main socio-economic activity is agriculture, with livestock ranking second to crop production. 

The three other regions are found in the northern belt of Ghana, occupying almost half of the land area of the country. The vegetation of the three regions is mainly savannah woodland with grassland and scattered drought-resistant trees such as baobab, acacia and shea tree, characterized by six months of a single rainy season with a prolonged dry, cold and hazy harmattan season. The three northern regions are popular with traditional poultry production and account for the majority of the local poultry found in the country [21].

### 2.2. Surveys

Data from two different surveys were used in the study. The first survey (hereafter named general survey) was a large-scale survey carried out to collect information on poultry farming in Ghana in the framework of the project “Insects as Feed in West Africa—IFWA”. A structured questionnaire was administered to 1960 farmers, randomly selected from 31 districts in the four study regions between October and December 2015. Most farmers were practicing indigenous poultry production but others had a more industrial production. Enumerators were obtained from the Regional Agricultural Departments, trained on the expectations of the study to assist in administering the questionnaires. The questionnaire included, among others, questions on the use of termites as feed to poultry, as well as characteristics of the interviewees. The questions on termites that were retained for this study were: the use of termites as poultry feed (possible answers: never, seldom, sometimes, often, always). The reasons for using termites or not were also collected. The questions that were used to analyze the factors affecting the use of termites were: region; sex; age (six groups of 10 years intervals); religion (Christian, Muslim, Traditional, others); educational background (none, non-formal, middle, junior high school, senior high school, tertiary, university); Annual income from poultry farming (in GHC); years of poultry farming (11 groups of five year intervals), farm size (ha). 

A second survey (hereafter named specific survey) was later conducted in selected villages to obtain more precise data on the practices of termite use as feed. Villages were randomly selected from districts that, from the general survey, most frequently used termites as feed for poultry. Since the general survey had shown that large, industrial poultry producers do not use termites, the specific survey focused on traditional poultry farmers. A questionnaire was designed that contained open questions on: termite species used (local name); description of termites used; collection or trapping methods; toxic termite species (local name); description of toxic species; reason for being termed toxic/poisonous; effect on the poultry when fed; variations in the termite species collected in the different seasons. The questionnaire of the specific survey is provided in Supplementary Material 1. The total number of farmers interviewed was 334. Samples of termite species mentioned by the farmers were collected and sent to the laboratory for taxonomic classification and analysis. 

### 2.3. Termite Identification

Termites were identified to the genus using Bouillon and Mathot [22] and Sands [23]. In addition, *Macrotermes* species were identified using Ruelle [24]. Only genera were included in the analyses, firstly because, in West Africa, most genera cannot be identified with certainty to the species level [25] and, secondly, because congeneric species usually had similar local names.

### 2.4. Data Analysis

Data from the general survey were used to analyze the factors affecting the use of termites as poultry feed by farmers. Two groups of poultry farmers were defined according to their use of termites. Those that answered “never” or “seldom” were classified as the non-users. Those that answered “sometimes”, “often” or “always” were classified as users. The effect of the different factors on the use of termites was examined using a binomial regression analysis. All factors were fixed: region; sex; age; religion; educational background; annual income from poultry farming; years of poultry farming; farm size. After the removal of respondents that had not correctly answered at least one of the questions, 1232 cases were included in the analyses. A stepwise selection of variables was first made before adjusting the model to the data, in order to avoid correlations between explanatory variables in the model. A stepwise regression incorporated both backward and forward approaches, and finally considered only significant explanatory variables in the final model. The selection of variables and the adjustment of the binomial regression to the data were carried out using R3.3.4 [26], with the generalized linear model function of the package vector generalized linear and additive models [27]. 

From the specific survey, we used the data of four regions of Ghana to build a termite species relative frequency matrix based on local people citation. Although the total number of the local sample of termite species surveyed in all the districts reached 105, after identification in the laboratory we enumerated only eight genera. Thus, matrix A (four regions and eight genera) was submitted to a principal component analysis (PCA) to describe the relationship between regions and termite genera.

Furthermore, we used the data from the four regions of Ghana to build a termite genera collection methods relative frequency matrix based on local people citation. The surveys had identified five collection methods and, thus, in the matrix B four regions and five methods were submitted to a principal component analysis (PCA) to describe the relationship between regions and termite species collection methods.

## 3. Results

### 3.1. Use of Termites as Feed in Ghana

The general survey showed that only 11% of the respondents said that they never give termites to their poultry in Ghana (Table 1). Twenty-three percent and 19% said that they “always” or “often” give termites, respectively. The most frequent answer was “sometimes”. The most commonly given reasons for giving termites were that it was healthy for poultry, good for their growth and an easily accessible source of protein. The main reasons for not giving termites were the lack of time and their scarcity, although some also mentioned health problems for poultry. In general, larger poultry breeders did not give termites because they used industrial feed and providing termites to a high number of poultry heads is unpractical. 

When assessing the factors significantly influencing the use of termites, the stepwise selection allowed us to select five factors: region, sex, education, farm size and income from poultry (Akaike information criterion = 1033.03 for the saturated model and 983.09 after the selection of the five factors). The detailed results of the binomial regression model (Table 2) showed that the region affected the use of termites. Male respondents were more likely to use termites. Higher education (tertiary and university) and income from poultry were negatively related to the use of termite, in contrast to farm size that was positively related to termite use. 

### 3.2. Termite Genera and Species 

The relative proportions of termite species cited by farmers according to the regions are shown in Table 3. More than 105 local termite names were recorded during the interviews of the specific survey, due to the fact that termite genera and species have different names in the different local languages, and that different names are often given for different cast members of same species. Generally, termites are described by their color, size and shape, nature and color of mound and type of feed eaten by termites. After the examination of samples, eight genera were identified and related to the local names (Supplementary Material 2): *Macrotermes*, *Odontotermes*, *Trinervitermes*, *Microtermes*, *Cubitermes*, *Allodontermes*, *Microcerotermes* and *Amitermes*. Samples of *Macrotermes* spp. were identified to species level and two species, *M. bellicosus* (Smeathman) and *M. subhyalinus* (Rambur) were found. 

In the specific survey, more than 90% of farmers in all regions cited the use of *Trinervitermes* species in feeding poultry (Table 3). The most frequently used species in the three regions in the north include species of the genera *Macrotermes*, *Odontotermes* and *Trinervitermes*. The use of *Cubitermes*, *Amitermes* and *Microtermes* was mentioned to a lesser extent. *Amitermes* was cited by more than 50% of the farmers in the Northern region but much less in other regions. There was no mention of *Odontotermes*, *Cubitermes* and *Microtermes* in the Volta region whereas *Allodontermes* and *Microcerotermes* were mentioned only in this region.

**Figure 1:**
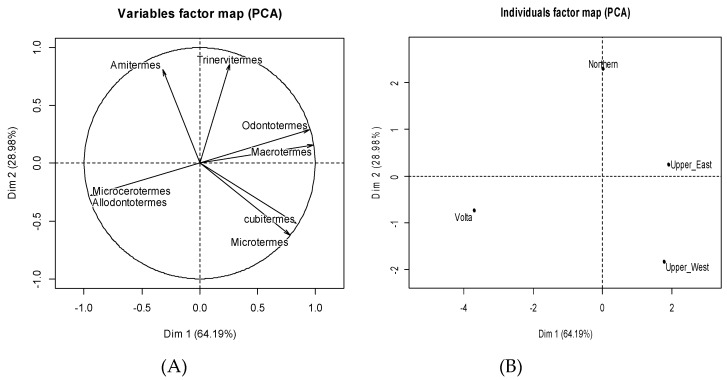
Principal component analysis results for the description of relationships between termite genera and regions. (**A**) correlation circle of termite genera. (**B**) projection of the regions in the first factorial plane formed by the axes 1 and 2 defined by the termite genera. Data from the specific survey.

The PCA analysis revealed that the termite genera *Macrotermes*, *Odontotermes*, *Microtermes* and *Cubitermes* are positively correlated with the first axis (Figure 1A) while the termite genera *Allodontermes* and *Microcerotermes* are negatively correlated with the same axis (Figure 1A). The second axis is positively correlated with the termite genera *Trinervitermes* and *Amitermes* and negatively with the termite genera *Microtermes* and *Cubitermes* (Figure 1A); these two first axes account for 93.16% of the variations in the data matrix. The projection of the regions in Axis 1 and 2 (Figure 1B) shows that, in general, termite of the genera *Macrotermes*, *Odontotermes*, *Microtermes* and *Cubitermes* were more cited in the Upper East and/or Upper West regions. On the other hand, termites of the less commonly cited genera *Allodontermes* and *Microcerotermes* were more cited in the Volta region. Finally, the *Amitermes* genus was more cited in the Northern region whereas *Trinervitermes* was abundantly used in all four regions.

### 3.3. Methods and Seasons of Collection 

Five methods of termite collection were identified during the specific survey. 

#### 3.3.1. Method 1

**Figure 2:**
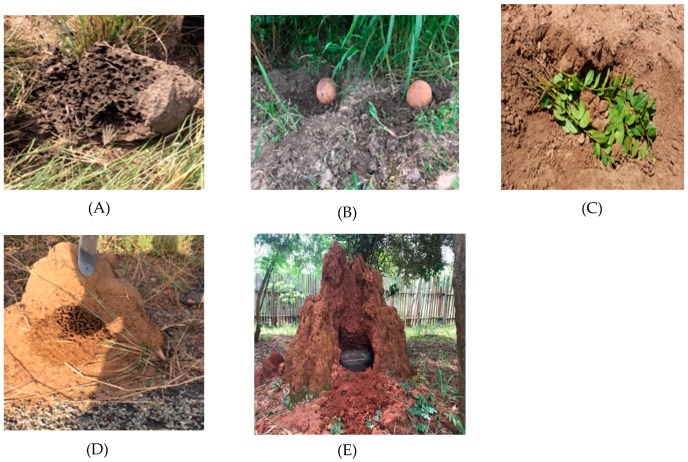
Collecting methods used in obtaining termites: (**A**) method 1; (**B**) method 2; (**C**) method 3; (**D**) method 4; (**E**) method 5.

Removal of whole or part of the mound. With the aid of an axe or hoe, the base around the mounds is dug to loosen it and the whole mound is removed. *Trinervitermes* species is harvested using this method because of the usually small size of their mounds (Figure 2A). Usually, a small hole is made on top of the mound and brushed with a branch to remove soldiers out of the way before digging out the mound because soldiers are known to be toxic. During the dry season, some farmers go at night to water mounds to enhance harvest in the morning. Although it seems the easiest method, it is destructive. The same termite mound can only be re-harvested after it has been rebuilt by the colony. 

#### 3.3.2. Method 2 

Inverting pot or container with organic matter over termite trails and small nests. This method is used mostly for harvesting *Odontotermes* and *Macrotermes* species (Figure 2B). The topsoil is removed to expose the tunnels and a pot filled with moistened organic matter is placed, inverted on the trail and collected after 12 h. Organic materials often used include dried cow dung, millet stalks, mango stones (seed), maize cobs/stalks, fresh leaves, groundnut husk/leaves, sorghum stalks, baobab fruits, donkey dung, watermelon parts, mahogany fruits/seeds, neem leaves, yam peels and banana leaves. The organic substrates in the pots are secured with stalks of millet, sorghum or flexible stems of trees to prevent them from falling when inverted and dried materials moistened with water. The common containers often used are earthen pots, open top gourd, old buckets and one liter open top jerry cans. The size of the container selected is dependent on the flock size of the farmer. The same trails and nests can be used for a long time and the method does not destroy the colony. Therefore, it is considered more sustainable than the other methods involving the destruction of the whole, or a part of the mound.

#### 3.3.3. Method 3

Filling a hole made in a mound with fresh leaves and collecting after a few hours, usually between 2–3 h (Figure 2C). Fresh branches with leaves from any plant are placed in a hole made in a *Macrotermes* mound, which is then covered with debris and left for between 2–3 h. Termite attracted to feed on the leaves are collected together with the leaves. 

#### 3.3.4. Method 4 

Removing part of the mound and collecting the newly rebuilt part (Figure 2D). A very simple procedure which involves removing part of a *Macrotermes* mound and returning after about 5 h to collect the newly rebuilt part of the mound, which is full of termites. 

#### 3.3.5. Method 5 

Fixing a basket with the debris of the mound in a hole made in the mound (Figure 2E). A hole is made in a *Macrotermes* termitarium until the farmer reaches the fungus garden (farmers identify fungus garden by their characteristic white color). A basket is placed in the hole created, debris collected into a basket and covered with leaves for 24 h. The basket is collected in the morning and the termites fed to birds. 

The targeted species and the size of the mound determine the collection method. Methods 3, 4 and 5 are used for collecting *Macrotermes* species because of their large mounds and the hardiness and difficulty of removing whole mounds. Termites with smaller mounds (e.g., *Trinervitermes* and *Cubitermes*) are typically collected using method 1 whereas termites with underground nests (e.g., *Odontotermes*) are trapped using method 2. However, this latter is also commonly used to trap *Macrotermes*. 

**Figure 3:**
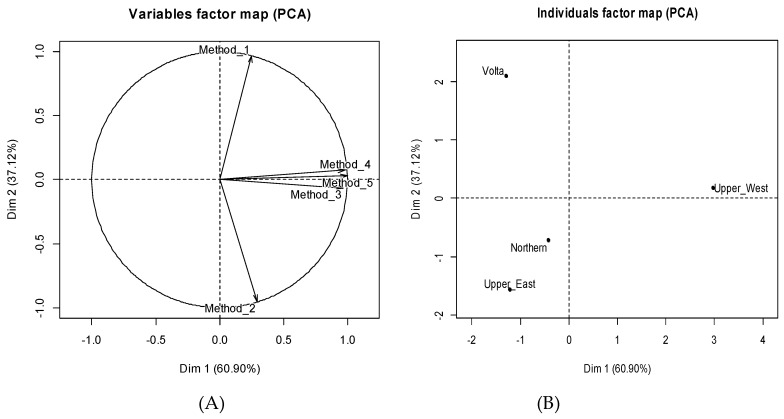
Principal component analysis results for the description of relationships between termite species collection methods and regions. (**A**) correlation circle of collection methods. (**B**) projection of the regions in the first factorial plane formed by axes 1 and 2 defined by the collection methods. Data from the specific survey.

**Figure 4:**
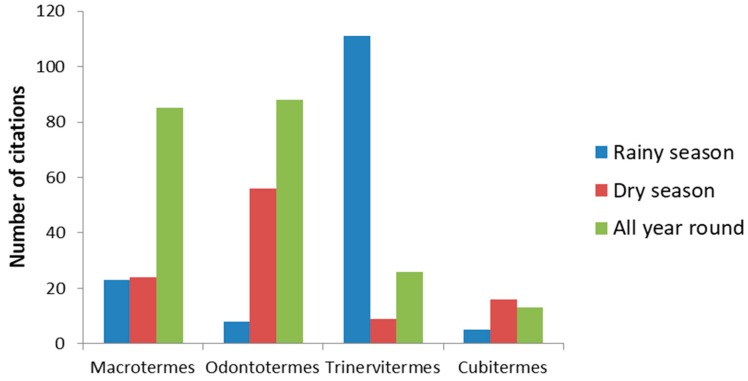
Association between the four main termite genera and season indicated as the number of citations by farmers. Data from the specific survey.

The results of the principal component analysis performed to describe the diversity of termite collection methods used by local populations in relation to the regions show that the factorial plan formed by axes 1 and 2 explains 98.2 % of the differences in the data matrix. The analysis of the correlations between these two axes and the methods allows us to deduce that methods 3, 4 and 5 have a positive correlation with the first axis (Figure 3A). The second axis is positively correlated with method 1 but negatively with method 2 (Figure 3A). The projection of the regions in the factorial plane formed by axes 1 and 2 (Figure 3B) shows that methods 3, 4 and 5 are much more used in the Upper West region for termite species collection. More specifically, method 1 is the only method mentioned in the Volta region while method 2 is frequently used in the other three regions and the dominant one in Upper East region and to a lesser extent in Northern region (Figure 4). The other three methods are rarely used except for method 3 in the Upper West region. A total of 76.6% of farmers interviewed in the specific survey use method 2 for collecting termites. The proportion of farmers using method 1, 3, 4, and 5 are 41.3%, 13.2%, 1.2%, and 2.4% respectively.

The termite genera collected depend on the seasons (X-squared = 217.17, df = 6, *p*-value < 2.2 × 10 ^−16^). *Trinervitermes* spp. are collected mostly in the rainy season (Figure 4). The other species are collected throughout the year although *Odontotermes* is more collected in the dry season.

### 3.4. Poisonous Termite Species

Results from interviews revealed that four types of termites were mentioned as unsuitable as feed, i.e., soldiers of both *Macrotermes* and *Trinervitermes*, some species of *Cubitermes*, and *Amitermes* (Table 4). Furthermore, it was reported that species with mounds built under Naudea latifolia Smith shrub locally called “gulungu” are also unsuitable. In addition, farmers were of the view that younger birds (less than four weeks old) were more susceptible to toxic species than older birds.

Farmers observed that feeding poisonous species may cause death in poultry. Other symptoms mentioned are weakness, dullness, paralysis, difficulty in excretion and reduced food intake. However, some of the symptoms farmers gave as the effects of poisoning on poultry were inconclusive.

## 4. Discussion

### 4.1. Use of Termites as Feed in Ghana

The information compiled from the present study revealed that the use of termites as feed for poultry is a very common practice in the four regions investigated. The proportions of farmers using termites found in this study are very similar to those observed in Burkina Faso, where Sankara et al. [20] showed that 78 % of the poultry farmers use termites to feed their birds at least occasionally. The use of termites was negatively correlated to education levels probably because farmers with higher education were those that had industrial or semi-industrial poultry farms and these are less suitable for the use of termites as protein feed. These farmers tend to stock a larger number of birds under intensive or semi-intensive systems and would require larger amounts of termites to feed their birds. Obtaining large quantities of termites to feed their birds would be difficult and unreliable by harvesting from the wild as this is the case now. 

### 4.2. Termite Genera and Species

Important variations were observed in the genera collected in the different regions. The genus most cited in all the regions is *Trinervitermes*, with more than 90% of all farmers citing this genus. Perhaps, *Trinervitermes* is used the most due to the abundance of their mound and the ease of obtaining workers by breaking mounds. Chrysostome [15] in Benin, and Diawara [19] in Burkina Faso, also cited the common use of *Trivervitermes* species as poultry feed. In Volta region, *Trinervitermes* species are by far the most commonly used termites, possibly due to their availability as the more humid climatic conditions may favour their abundance, compared to the Northern region, which experiences just one rainy season in the year. The unfamiliarity with use of *Odontotermes* by Volta region farmers raises some curiosity as farmers in Northern Ghana mentioned that *Odontotermes* is the best termite to use as feed since no cast member is known to cause any toxicity to poultry when consumed. An investigation needs to be carried out to verify whether the absence of *Odontotermes* in poultry diet in the Volta region is due to its low abundance or to a lack of knowledge in harvesting techniques.

Farmers in the three northern regions cited the use of *Macrotermes* more frequently than in the Volta region. Farmers in the Volta region are aware of their use but collect them by breaking mounds and seem to be poorly aware of the trapping techniques used in the northern parts of the country. *Macrotermes bellicosus,* the species most commonly found in our samples, is probably the most cited termite species used as human food or livestock diet, in many countries [1,28]. *Macrotermes* species are also a delicacy for gorillas and chimpanzees [29]. The preference or special liking for *Macrotermes* may be attributed to its large size and the abundance of the genus in Africa [1]. 

### 4.3. Methods and Seasons of Collection

Five termite collection methods were reported. While their respective use depends on the targeted termite species and region, the two most common methods were Method 1 (breaking the mounds) and Method 2 (inverted container). These methods are also the most commonly used in Burkina Faso [19,30,31]. Different termite species are collected in different seasons of the year. *Trinervitermes* is collected mainly in the rainy season, probably because mounds are broken more easily during the rain. *Macrotermes* and *Odontotermes* are collected throughout the year, although collecting this latter seems more common in the dry season. *Odontotermes* is collected mainly by trapping with baited inverted pots (Method 2) and this method is particularly well adapted to the dry season since, in the rainy season, termite tracks are usually washed off by the rain.

Harvesting and trapping methods depend on the termite species of interest to the farmer. For all species, harvesting and collection are usually done in the early hours of the morning (between 6 am–8 am) as termites move deeper into mounds during the hot afternoons. *Macrotermes* and *Odontotermes* are mainly collected using baits and elaborate trapping methods in contrast to *Trinervitermes*, which are collected by removing the mound, thereby destroying the whole mound in process. Farina et al. [16] and Chrysostome et al. [18] described similar trapping methods with bait pots, used by poultry farmers in Togo and Benin in harvesting termites to feed poultry. Time for regeneration of mound after its removal is unknown, although, as recognized by farmers, if the queen is not removed, new mounds will definitely be rebuilt. Complete removal of mounds is unsustainable and therefore needs to be discouraged. 

Containers of various sizes, shapes and material are used for trapping termites when using the baited container technique. The size of the container is usually dependent on the size and age of flock, larger and younger flock requiring bigger containers as more termites are needed in feeding. Containers are usually made from earthenware, plastic, calabash or metal material; however, farmers advise the use of earthenware as it remains cooler than containers made of other materials. To maintain good microclimate for the termites, baited containers can be covered with leaves after placing on termite mounds. 

### 4.4. Poisonous Termite Species

Farmers described several termite species or castes as being poisonous or damaging for their poultry. However, the mechanisms of toxicity would need to be investigated and verified through specific studies. Poisonous species described by farmers include *Amitermes* and *Cubitermes* species and the soldier caste of *Trinervitermes* and *Macrotermes* species. In most cases, it was mentioned that only chicks and keets were vulnerable. Prestwich [32] described the chemical mechanisms used by termites in defense against intruders of their mound. The mechanisms deployed are: biting mouthpart with simultaneous injection of toxins; irritants or oily chemical secretions from frontal gland; application of a topically active poison using the labrum; ejection of viscous sticky solution which irritates and mechanically immobilizes small assailants. *Amitermes* and *Macrotermes* bite and inject toxins while *Trinervitermes* secrete toxins and irritants. Farmers observed that *Macrotermes* soldiers kill by biting the throat of chicks. They also suggest that the death of poultry fed on soldiers of *Trinervitermes* are caused by the secretion of sticky substance by soldiers. Indeed, the toxicity of *Trinervitermes* species, also mentioned by Diawara [19] is most likely due to the diterpenes and monoterpenes that are released from their snout to deter ants and predators [33,34,35]. 

Chicks and keets’ throats are probably more sensitive to bites and they may also have an underdeveloped immune system. However, farmers also described various symptoms of illness caused by toxic termites (Table 4) and the causes and mechanisms involved in the reported death or symptom of illness following the consumption of certain termite species. 

Farmers have developed techniques to avoid the collection of a high quantity of soldiers. When collecting *Trinervitermes* from mounds, a small hole is first made on the mound causing soldiers to come out to attack. The soldiers are teased out with a branch after which the part containing a majority of workers are removed. Furthermore, farmers avoid collecting *Trinervitermes* from old mounds and believe that new mounds containing more grass are less poisonous because workers are comparatively more abundant in such mounds. The adverse effect of *Trinervitermes* may be reversed, when observed, early by soaking bitter leaves of *Vernonia amygdalina* in water and give to the bird to drink. 

To avoid injury to chicks and keets, *Macrotermes* is usually given to birds older than 1 or 1.5 months. *Cubitermes* is said to be usually poisonous in the dry season, but when fed to birds 4 weeks after hatching, causes no adverse effect. 

No toxicity of *Odontotermes* was cited. Farmer communication indicated that among the termite species collected and used, the safest and most nutritious is *Odontotermes*, supporting growth and wellbeing of poultry. 

## 5. Conclusions

Farmers consider termites as very important because they are an easily available protein for poultry. However, the collection of termite mounds is difficult because their availability varies with season and, in some cases, farmers must walk long distances to obtain them. In addition, mounds’ collection is destructive and should not be encouraged. Therefore, trapping techniques should be promoted and the existing methods should be improved. In regions where trapping is uncommon, e.g., Volta Region, investigations should be made to find out if the termites suitable for being trapped (*Macrotermes* and *Odontotermes*) are sufficiently abundant to encourage the adoption of trapping methods. The issue of toxicity of some termites needs to be verified, including the cause and mechanisms of toxicity, the toxins responsible for toxicity and methods to avoid the harvesting of toxic species and castes. Some symptoms mentioned relate to common poultry diseases and therefore must be verified. The nutritional profile of the genera should be studied to ascertain the assertion by farmers that the most nutritious is *Odontotermes*, 

## Figures and Tables

**Table 1:** In the general survey, the frequency of responses (%) of farmers from the four regions to the question: Do you use termites to feed your poultry? (number of farmers = 1817).

Region	Never	Seldom	Sometimes	Often	Always
Northern	21.81	2.70	50.00	11.27	14.22
Upper East	5.54	4.48	35.18	29.21	25.59
Upper West	7.05	2.42	39.21	29.74	21.59
Volta	9.69	8.45	47.01	6.60	28.25
**Total**	**10.68**	**4.63**	**42.68**	**19.27**	**22.74**

**Table 2:** Results of the binomial linear regression. Probability values that are significant at 0.05 level are in bold.

Factors	Estimate	Std. Error	Probability
(Intercept)	0.610	0.356	>0.05
Region—Upper East	1.517	0.250	**<0.001**
Region—Upper West	1.551	0.266	**<0.001**
Region—Volta	0.780	0.235	**<0.001**
Sex—male	0.483	0.232	**<0.05**
Education—middle	−0.307	0.335	>0.05
Education—none	−0.073	0.255	>0.05
Education—non-formal	−0.119	0.551	>0.05
Education—senior high school	−0.199	0.357	>0.05
Education—tertiary	−0.958	0.371	**<0.01**
Education—university	−1.881	0.404	**<0.001**
Farm size	0.077	0.033	**<0.05**
Income from poultry	−0.00024	0.00008	**<0.01**

**Table 3:** In the specific survey, the frequency of eight termite genera mentioned by farmers as feed for poultry in the four regions (number of farmers = 334).

Region	*Trinervi-termes*	*Macro-termes*	*Odonto-termes*	*Ami-termes*	*Micro-termes*	*Cubi-termes*	*Allodon-termes*	*Microcero-termes*
Upper West	94.4	83.0	71.9	10.1	25.8	51.7	0.0	0.0
Northern	96.8	76.6	74.4	58.5	0.0	10.6	0.0	0.0
Upper East	96.9	92.3	99.2	7.7	17.7	30.0	0.0	0.0
Volta	95.0	40.0	0.0	25.0	0.0	0.0	10.0	10.0

**Table 4:** Termite genera mentioned by farmers as toxic or poisonous and symptoms of illness, usually only in chicks and keets.

Termites	Symptoms
Soldiers of *Macrotermes*	Death due to choking
*Cubitermes* (some species)	Dizziness, weakness, reduced food intake (weight loss), diarrhea, indigestion, and death
Soldiers of *Trinervitermes*	Dullness, dizziness, weakness, paralysis and death
*Amitermes*	Difficulty in excretion and death

## Supplementary Materials

The following are available online at https://www.mdpi.com/2075-4450/10/3/69/s1. Supplementary Material 1: Questionnaire of the specific survey; Supplementary Material 2: Phonetic transcriptions of termite genera in the four investigated regions.
