# Supplementary material for: Use of Termites by Farmers as Poultry Feed in Ghana
Source: Insects. 2019 Mar 13;10(3):69. doi: 10.3390/insects10030069 (PMC6468897; doi:10.3390/insects10030069)
Supplement: Supplementary file 1 [file insects-10-00069-s001.zip › Supplementary material 2.docx]

| Supplementary material 2. Phonetic transcriptions of termite genera in the four investigated regions  To  Use of termites by farmers as poultry feed in Ghana  Boafo, Hettie Arwoh, Affedzie-Obresi, Siegfried, Gbemavo, Dossou Séblodo Judes Charlemagne, Clottey, Victor Attuquaye, Nkegbe, Emmanuel, Adu-Aboagye, Gabriel and Kenis, Marc  Table S2. Phonetic transcriptions of termite genera in the four investigated regions | | | |
| --- | --- | --- | --- |
| **Region** | **Local name** | **Genus name** | **Caste** |
| Volta |  |  |  |
|  | Babanoe | *Trinervitermes* | Workers |
|  | Babataga | *Macrotermes* | All members |
|  | Babatsifome | *Microtermes* | All members |
|  | Babatsoe | *Trinervitermes* | Soldiers |
|  | Babanulabe | *Trinervitermes* | Soldiers |
|  | Babanto | *Trinervitermes* | Soldiers |
|  | Babasoe | *Trinervitermes* | Workers |
|  | Babagye | *Trinervitermes* | Soldiers |
|  | Babayentor | *Trinervitermes* | Soldiers |
| Upper West |  |  |  |
|  | Kpele/Kpolo | *Odontotermes* | All members |
|  | Tambiezie | *Trinervitermes* | Soldiers |
|  | Zuzie | *Trinervitermes* | Soldiers |
|  | Kontontambire | *Trinivertermes* | Soldiers |
|  | Moree | *Odontotermes* | All members |
|  | Guno | *Cubitermes* | All members |
|  | Yawzugboli | *Macrotermes* | Soldiers |
|  | Yawmaa | *Macrotermes* | Queen |
|  | Zugboli | *Macrotermes* | Soldiers |
|  | Dadigre | *Cubitermes* | All members |
|  | Dadiga | *Cubitermes* | All members |
|  | Yaokpele | *Trinervitermes* | Workers |
|  | Feeli | *Trinervitermes* | Soldiers |
|  | Tien | *Macrotermes* | All members |
|  | Feelidjuron | *Cubitermes* | All caste |
|  | Feelikokoo | *Trinervitermes* | Soldiers |
|  | Tambituo | *Trinervitermes* | Soldiers |
| Upper East |  |  |  |
|  | Kunfio/fio | *Trinervitermes* | Soldiers |
|  | Kunkwio | *Macrotermes* | All members |
|  | Tua/Toa | *Microtermes* | All members |
|  | Morka | *Odontotermes* | All members |
|  | Kotonko | *Trinervitermes* | Soldiers |
| Northern |  |  |  |
|  | Tambiezie | *Trinervitermes* | Soldiers |
|  | Mochereba | *Trinervitermes* | Workers |
|  | Tambie pielegu | *Amitermes* | All members |
|  | Worikogu | *Odontotermes* | All members |
|  | Tambiegun | *Amitermes* | All members |
|  | Tambietuo | *Trinervitermes* | Soldiers |
|  | Tambie gbungara | *Cubitermes* | All members |
|  | Yoblezie | *Macrotermes* | Soldiers |
|  | Gbutegba | *Trinervitermes* | Soldiers |
