# Supplementary material for: Use of Termites by Farmers as Poultry Feed in Ghana
Source: Insects. 2019 Mar 13;10(3):69. doi: 10.3390/insects10030069 (PMC6468897; doi:10.3390/insects10030069)
Supplement: Supplementary file 1 [file insects-10-00069-s001.zip › Supplementary material 1.docx]

Supplementary material 1. Questionnaire of the specific survey

To

Use of termites by farmers as poultry feed in Ghana

Boafo, Hettie Arwoh, Affedzie-Obresi, Siegfried, Gbemavo, Dossou Séblodo Judes Charlemagne, Clottey, Victor Attuquaye, Nkegbe, Emmanuel, Adu-Aboagye, Gabriel and Kenis, Marc

**IFWA TERMITE SURVEY (GHANA)**

**Sample Data Sheet**

**Name of Enumerator/ Scientist**………………………………. **Date**…………………

**GPS**………….……..

|  | Review |
| --- | --- |

1. Village/Town

|  |
| --- |

2. Name of Farmer

|  |
| --- |

3. Telephone

|  | Sample ID |
| --- | --- |

4. Termite species used

|  |
| --- |

5. Description of termites used

|  |
| --- |

6. Collection or trapping methods

|  |
| --- |

7. Toxic termite species

|  | Sample ID |
| --- | --- |

8. Description of toxic species

|  |
| --- |

9. Reason for being

termed toxic/

poisonous

|  |
| --- |

10. Any effect on the poultry when fed?

|  |
| --- |

11. Are there any

variations in the termite species collected

in the different seasons?
